# 2,6-Bis(2,4-dichloro­benzyl­idene)cyclo­hexa­none

**DOI:** 10.1107/S1600536808023003

**Published:** 2008-07-31

**Authors:** Huan-Mei Guo, Li Liu, Fang-Fang Jian

**Affiliations:** a Microscale Science Institute, Department of Chemistry and Chemical Engineering, Weifang University, Weifang 261061, People’s Republic of China

## Abstract

The title compound, C_20_H_14_Cl_4_O, was prepared from a mixture of 2,4-dichloro­benzophenone and cyclo­hexa­none. The dihedral angles formed by  the cyclohexane ring  and two benzene rings are 39.18 (2) and 60.72 (2)°. There are some weak intra­molecular C—H⋯O and C—H⋯Cl hydrogen-bond contacts in the crystal structure.

## Related literature

For related literature, see: Butcher *et al.* (2006 ▶); Deli *et al.* (1984 ▶); Jia *et al.* (1989 ▶); Yu *et al.* (2000 ▶).
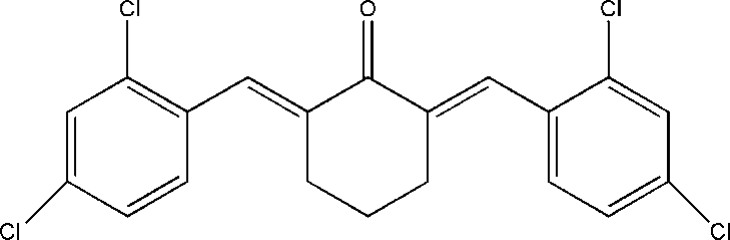


## Experimental

### 

#### Crystal data

C_20_H_14_Cl_4_O
*M* *_r_* = 412.11
Orthorhombic, 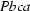

*a* = 14.469 (2) Å
*b* = 8.0602 (12) Å
*c* = 31.554 (4) Å
*V* = 3679.9 (9) Å^3^
*Z* = 8
Mo *K*α radiation
μ = 0.65 mm^−1^
*T* = 293 (2) K
0.20 × 0.15 × 0.10 mm

#### Data collection

Bruker SMART CCD area-detector diffractometer
Absorption correction: multi-scan (*SADABS*; Bruker, 1997 ▶) *T*_min_ = 0.881, *T*_max_ = 0.938
21985 measured reflections
4570 independent reflections
2735 reflections with *I* > 2σ(*I*)
*R*_int_ = 0.053

#### Refinement

*R*[*F* ^2^ > 2σ(*F* ^2^)] = 0.050
*wR*(*F* ^2^) = 0.118
*S* = 1.03
4570 reflections
226 parameters
H-atom parameters constrained
Δρ_max_ = 0.24 e Å^−3^
Δρ_min_ = −0.28 e Å^−3^

### 

Data collection: *SMART* (Bruker, 1997 ▶); cell refinement: *SAINT* (Bruker, 1997 ▶); data reduction: *SAINT*; program(s) used to solve structure: *SHELXS97* (Sheldrick, 2008 ▶); program(s) used to refine structure: *SHELXL97* (Sheldrick, 2008 ▶); molecular graphics: *SHELXTL* (Sheldrick, 2008 ▶); software used to prepare material for publication: *SHELXTL*.

## Supplementary Material

Crystal structure: contains datablocks I, global. DOI: 10.1107/S1600536808023003/at2592sup1.cif

Structure factors: contains datablocks I. DOI: 10.1107/S1600536808023003/at2592Isup2.hkl

Additional supplementary materials:  crystallographic information; 3D view; checkCIF report

## Figures and Tables

**Table 1:** Hydrogen-bond geometry (Å, °)

*D*—H⋯*A*	*D*—H	H⋯*A*	*D*⋯*A*	*D*—H⋯*A*
C7—H7*A*⋯Cl2	0.93	2.74	3.055 (2)	101
C7—H7*A*⋯O1	0.93	2.33	2.732 (3)	105
C14—H14*A*⋯O1	0.93	2.39	2.759 (3)	103

**Fig. 1.**
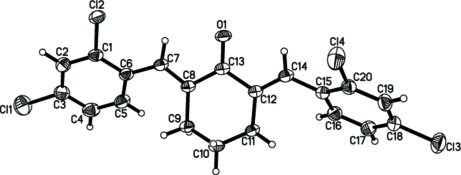
The structure of the title compound, showing 30% probability displacement ellipsoids and the atom-numbering scheme.

## supplementary crystallographic information

### Comment

As useful precursors to potentially bioactive pyrimidine derivatives, a,a-bis(substituted benzylidene) cycloalkanones have attracted considerable attention for many years (Deli *et al.*, 1984). In recent years, a series of non-linear optically active bis(benzylidene) ketones have been synthesized (Yu *et al.*, 2000). As part of our search for new non-linear optically active compounds, we synthesized the title compound (I), and describe its structure here.

In the structure of (I) (Fig. 1), all of the bond lengthes and bond angles fall in the normal range (Yu *et al.*, 2000; Jia *et al.*, 1989; Butcher *et al.*, 2006). There are some weak C—H···O and C—H···Cl intramolecular hydrogen bonds in the crystal structure.

### Experimental

A mixture of the 2,4-dichlorobenzophenone (0.2 mol), and cyclohexanone (0.1 mol) and 10% NaOH (10 ml) was stirred in ethanol (30 mL) for 5 h to afford the title compound [yield: 82%]. Single crystals suitable for X-ray measurements were obtained by recrystallization from ethanol at room temperature.

### Refinement

H atoms were fixed geometrically and allowed to ride on their attached atoms, with C—H = 0.93–0.97 Å, and with *U*_iso_(H) = 1.2*U*_eq_(C).

### Crystal data

**Table:**

C_20_H_14_Cl_4_O	*Z* = 8
*M**_r_* = 412.11	*F*_000_ = 1680
Orthorhombic, *P**b**c**a*	*D*_x_ = 1.488 Mg m^−^^3^
Hall symbol: -P 2ac 2ab	Mo *K*α radiation λ = 0.71073 Å
*a* = 14.469 (2) Å	µ = 0.65 mm^−^^1^
*b* = 8.0602 (12) Å	*T* = 293 (2) K
*c* = 31.554 (4) Å	Bar, yellow
*V* = 3679.9 (9) Å^3^	0.20 × 0.15 × 0.10 mm

### Data collection

**Table:**

Bruker SMART CCD area-detector diffractometer	4570 independent reflections
Radiation source: fine-focus sealed tube	2735 reflections with *I* > 2σ(*I*)
Monochromator: graphite	*R*_int_ = 0.053
*T* = 293(2) K	θ_max_ = 28.4º
φ and ω scans	θ_min_ = 1.3º
Absorption correction: multi-scan(SADABS; Bruker, 1997)	*h* = −19→18
*T*_min_ = 0.881, *T*_max_ = 0.938	*k* = −10→10
21985 measured reflections	*l* = −19→42

### Refinement

**Table:**

Refinement on *F*^2^	Secondary atom site location: difference Fourier map
Least-squares matrix: full	Hydrogen site location: inferred from neighbouring sites
*R*[*F*^2^ > 2σ(*F*^2^)] = 0.050	H-atom parameters constrained
*wR*(*F*^2^) = 0.118	*w* = 1/[σ^2^(*F*_o_^2^) + (0.0381*P*)^2^ + 1.6278*P*] where *P* = (*F*_o_^2^ + 2*F*_c_^2^)/3
*S* = 1.03	(Δ/σ)_max_ = 0.001
4570 reflections	Δρ_max_ = 0.24 e Å^−^^3^
226 parameters	Δρ_min_ = −0.28 e Å^−^^3^
Primary atom site location: structure-invariant direct methods	Extinction correction: none

### Special details

**Table:**

Geometry. All e.s.d.'s (except the e.s.d. in the dihedral angle between two l.s. planes) are estimated using the full covariance matrix. The cell e.s.d.'s are taken into account individually in the estimation of e.s.d.'s in distances, angles and torsion angles; correlations between e.s.d.'s in cell parameters are only used when they are defined by crystal symmetry. An approximate (isotropic) treatment of cell e.s.d.'s is used for estimating e.s.d.'s involving l.s. planes.
Refinement. Refinement of *F*^2^ against ALL reflections. The weighted *R*-factor *wR* and goodness of fit *S* are based on *F*^2^, conventional *R*-factors *R* are based on *F*, with *F* set to zero for negative *F*^2^. The threshold expression of *F*^2^ > σ(*F*^2^) is used only for calculating *R*-factors(gt) *etc*. and is not relevant to the choice of reflections for refinement. *R*-factors based on *F*^2^ are statistically about twice as large as those based on *F*, and *R*- factors based on ALL data will be even larger.

### Fractional atomic coordinates and isotropic or equivalent isotropic displacement parameters (Å^2^)

**Table:**

	*x*	*y*	*z*	*U*_iso_*/*U*_eq_	
Cl1	−0.37152 (7)	0.07869 (15)	−0.49524 (3)	0.1044 (4)	
Cl2	−0.51233 (4)	0.04759 (9)	−0.33808 (2)	0.05594 (19)	
Cl3	0.06595 (6)	0.60906 (12)	−0.04572 (3)	0.0852 (3)	
Cl4	−0.21635 (6)	0.20911 (12)	−0.08713 (2)	0.0825 (3)	
O1	−0.36351 (10)	0.3253 (2)	−0.22630 (5)	0.0557 (5)	
C1	−0.42738 (15)	0.1234 (3)	−0.37207 (8)	0.0468 (6)	
C2	−0.43315 (18)	0.0787 (4)	−0.41435 (8)	0.0578 (7)	
H2A	−0.4804	0.0098	−0.4238	0.069*	
C3	−0.36783 (19)	0.1381 (4)	−0.44228 (9)	0.0635 (8)	
C4	−0.29917 (19)	0.2442 (4)	−0.42879 (9)	0.0663 (8)	
H4A	−0.2572	0.2881	−0.4481	0.080*	
C5	−0.29343 (17)	0.2844 (4)	−0.38653 (9)	0.0592 (7)	
H5A	−0.2465	0.3550	−0.3776	0.071*	
C6	−0.35581 (16)	0.2229 (3)	−0.35643 (8)	0.0466 (6)	
C7	−0.34846 (15)	0.2620 (3)	−0.31106 (8)	0.0461 (6)	
H7A	−0.4041	0.2752	−0.2967	0.055*	
C8	−0.27162 (14)	0.2811 (3)	−0.28761 (8)	0.0419 (6)	
C9	−0.17413 (14)	0.2600 (3)	−0.30424 (8)	0.0499 (6)	
H9A	−0.1571	0.3581	−0.3202	0.060*	
H9B	−0.1727	0.1661	−0.3235	0.060*	
C10	−0.10328 (15)	0.2324 (3)	−0.26939 (8)	0.0502 (6)	
H10A	−0.1142	0.1261	−0.2559	0.060*	
H10B	−0.0417	0.2310	−0.2815	0.060*	
C11	−0.11002 (15)	0.3693 (3)	−0.23677 (8)	0.0457 (6)	
H11A	−0.0612	0.3563	−0.2160	0.055*	
H11B	−0.1020	0.4760	−0.2505	0.055*	
C12	−0.20266 (14)	0.3642 (3)	−0.21491 (8)	0.0412 (5)	
C13	−0.28552 (15)	0.3230 (3)	−0.24158 (7)	0.0423 (6)	
C14	−0.21556 (16)	0.3858 (3)	−0.17333 (8)	0.0466 (6)	
H14A	−0.2751	0.3680	−0.1632	0.056*	
C15	−0.14545 (15)	0.4349 (3)	−0.14198 (8)	0.0443 (6)	
C16	−0.08247 (16)	0.5614 (3)	−0.15088 (8)	0.0512 (6)	
H16A	−0.0840	0.6112	−0.1775	0.061*	
C17	−0.01834 (18)	0.6150 (3)	−0.12186 (8)	0.0554 (7)	
H17A	0.0227	0.6995	−0.1288	0.067*	
C18	−0.01524 (17)	0.5426 (3)	−0.08242 (8)	0.0539 (7)	
C19	−0.07653 (18)	0.4177 (3)	−0.07173 (8)	0.0555 (7)	
H19A	−0.0746	0.3691	−0.0450	0.067*	
C20	−0.14056 (17)	0.3665 (3)	−0.10137 (8)	0.0490 (6)	

### Atomic displacement parameters (Å^2^)

**Table:**

	*U*^11^	*U*^22^	*U*^33^	*U*^12^	*U*^13^	*U*^23^
Cl1	0.1071 (7)	0.1580 (10)	0.0482 (5)	−0.0068 (7)	0.0000 (5)	−0.0108 (5)
Cl2	0.0381 (3)	0.0687 (4)	0.0610 (4)	−0.0027 (3)	0.0010 (3)	0.0002 (3)
Cl3	0.0747 (5)	0.1149 (7)	0.0660 (5)	−0.0291 (5)	−0.0131 (4)	−0.0144 (5)
Cl4	0.0959 (6)	0.0945 (6)	0.0572 (5)	−0.0482 (5)	−0.0024 (4)	0.0122 (4)
O1	0.0313 (9)	0.0847 (14)	0.0509 (10)	−0.0009 (8)	0.0073 (8)	0.0041 (10)
C1	0.0355 (13)	0.0554 (16)	0.0497 (15)	0.0080 (11)	−0.0013 (11)	0.0045 (12)
C2	0.0506 (16)	0.0682 (19)	0.0546 (17)	0.0054 (13)	−0.0080 (13)	−0.0023 (14)
C3	0.0559 (17)	0.090 (2)	0.0445 (15)	0.0099 (16)	−0.0043 (14)	−0.0017 (15)
C4	0.0524 (16)	0.093 (2)	0.0531 (17)	0.0033 (16)	0.0063 (14)	0.0141 (16)
C5	0.0459 (15)	0.077 (2)	0.0549 (17)	−0.0076 (13)	−0.0001 (13)	0.0080 (15)
C6	0.0355 (12)	0.0555 (15)	0.0488 (15)	0.0069 (11)	−0.0019 (11)	0.0027 (12)
C7	0.0335 (12)	0.0547 (15)	0.0501 (15)	0.0002 (10)	0.0026 (11)	0.0034 (12)
C8	0.0331 (12)	0.0459 (14)	0.0468 (14)	−0.0012 (10)	0.0041 (10)	0.0056 (11)
C9	0.0331 (12)	0.0648 (17)	0.0517 (15)	−0.0026 (11)	0.0061 (11)	−0.0020 (13)
C10	0.0318 (12)	0.0576 (16)	0.0612 (16)	0.0049 (11)	0.0045 (12)	−0.0007 (13)
C11	0.0316 (12)	0.0537 (15)	0.0518 (15)	−0.0006 (11)	−0.0006 (11)	0.0023 (12)
C12	0.0327 (12)	0.0436 (14)	0.0473 (14)	0.0007 (10)	0.0024 (11)	0.0048 (11)
C13	0.0331 (12)	0.0458 (14)	0.0481 (14)	0.0018 (10)	0.0034 (11)	0.0096 (11)
C14	0.0340 (12)	0.0552 (15)	0.0507 (15)	−0.0003 (11)	0.0016 (11)	0.0050 (12)
C15	0.0370 (12)	0.0527 (15)	0.0431 (14)	0.0021 (11)	0.0027 (11)	−0.0019 (12)
C16	0.0490 (14)	0.0575 (16)	0.0471 (15)	−0.0007 (12)	0.0020 (12)	0.0047 (13)
C17	0.0513 (15)	0.0566 (17)	0.0583 (17)	−0.0101 (13)	0.0042 (13)	−0.0005 (14)
C18	0.0484 (14)	0.0633 (17)	0.0499 (16)	−0.0018 (13)	−0.0033 (12)	−0.0111 (14)
C19	0.0629 (17)	0.0642 (18)	0.0393 (14)	−0.0059 (14)	−0.0008 (12)	−0.0001 (13)
C20	0.0487 (14)	0.0513 (16)	0.0469 (15)	−0.0062 (12)	0.0052 (12)	−0.0013 (12)

### Geometric parameters (Å, °)

**Table:**

Cl1—C3	1.739 (3)	C9—H9B	0.9700
Cl2—C1	1.742 (2)	C10—C11	1.512 (3)
Cl3—C18	1.735 (3)	C10—H10A	0.9700
Cl4—C20	1.736 (3)	C10—H10B	0.9700
O1—C13	1.227 (2)	C11—C12	1.508 (3)
C1—C2	1.384 (3)	C11—H11A	0.9700
C1—C6	1.400 (3)	C11—H11B	0.9700
C2—C3	1.378 (4)	C12—C14	1.337 (3)
C2—H2A	0.9300	C12—C13	1.502 (3)
C3—C4	1.378 (4)	C14—C15	1.471 (3)
C4—C5	1.375 (4)	C14—H14A	0.9300
C4—H4A	0.9300	C15—C16	1.396 (3)
C5—C6	1.401 (3)	C15—C20	1.397 (3)
C5—H5A	0.9300	C16—C17	1.373 (3)
C6—C7	1.470 (3)	C16—H16A	0.9300
C7—C8	1.345 (3)	C17—C18	1.375 (4)
C7—H7A	0.9300	C17—H17A	0.9300
C8—C13	1.504 (3)	C18—C19	1.383 (4)
C8—C9	1.515 (3)	C19—C20	1.380 (3)
C9—C10	1.520 (3)	C19—H19A	0.9300
C9—H9A	0.9700		
			
C2—C1—C6	122.2 (2)	H10A—C10—H10B	108.2
C2—C1—Cl2	117.4 (2)	C12—C11—C10	110.4 (2)
C6—C1—Cl2	120.4 (2)	C12—C11—H11A	109.6
C3—C2—C1	119.0 (3)	C10—C11—H11A	109.6
C3—C2—H2A	120.5	C12—C11—H11B	109.6
C1—C2—H2A	120.5	C10—C11—H11B	109.6
C2—C3—C4	120.8 (3)	H11A—C11—H11B	108.1
C2—C3—Cl1	119.9 (2)	C14—C12—C13	117.9 (2)
C4—C3—Cl1	119.3 (2)	C14—C12—C11	124.7 (2)
C5—C4—C3	119.3 (3)	C13—C12—C11	117.3 (2)
C5—C4—H4A	120.3	O1—C13—C12	120.7 (2)
C3—C4—H4A	120.3	O1—C13—C8	120.4 (2)
C4—C5—C6	122.4 (3)	C12—C13—C8	118.93 (19)
C4—C5—H5A	118.8	C12—C14—C15	126.8 (2)
C6—C5—H5A	118.8	C12—C14—H14A	116.6
C1—C6—C5	116.1 (2)	C15—C14—H14A	116.6
C1—C6—C7	121.3 (2)	C16—C15—C20	116.1 (2)
C5—C6—C7	122.5 (2)	C16—C15—C14	120.7 (2)
C8—C7—C6	128.4 (2)	C20—C15—C14	123.1 (2)
C8—C7—H7A	115.8	C17—C16—C15	122.5 (2)
C6—C7—H7A	115.8	C17—C16—H16A	118.8
C7—C8—C13	116.5 (2)	C15—C16—H16A	118.8
C7—C8—C9	124.5 (2)	C16—C17—C18	119.5 (2)
C13—C8—C9	118.96 (19)	C16—C17—H17A	120.3
C8—C9—C10	113.2 (2)	C18—C17—H17A	120.3
C8—C9—H9A	108.9	C17—C18—C19	120.6 (2)
C10—C9—H9A	108.9	C17—C18—Cl3	119.7 (2)
C8—C9—H9B	108.9	C19—C18—Cl3	119.8 (2)
C10—C9—H9B	108.9	C20—C19—C18	118.9 (2)
H9A—C9—H9B	107.8	C20—C19—H19A	120.6
C11—C10—C9	110.0 (2)	C18—C19—H19A	120.6
C11—C10—H10A	109.7	C19—C20—C15	122.5 (2)
C9—C10—H10A	109.7	C19—C20—Cl4	117.9 (2)
C11—C10—H10B	109.7	C15—C20—Cl4	119.59 (19)
C9—C10—H10B	109.7		
			
C6—C1—C2—C3	−1.9 (4)	C11—C12—C13—O1	−176.1 (2)
Cl2—C1—C2—C3	179.2 (2)	C14—C12—C13—C8	−173.9 (2)
C1—C2—C3—C4	−1.9 (4)	C11—C12—C13—C8	3.0 (3)
C1—C2—C3—Cl1	178.0 (2)	C7—C8—C13—O1	5.7 (3)
C2—C3—C4—C5	3.2 (4)	C9—C8—C13—O1	−173.0 (2)
Cl1—C3—C4—C5	−176.7 (2)	C7—C8—C13—C12	−173.4 (2)
C3—C4—C5—C6	−0.8 (4)	C9—C8—C13—C12	7.9 (3)
C2—C1—C6—C5	4.1 (4)	C13—C12—C14—C15	−176.6 (2)
Cl2—C1—C6—C5	−176.99 (19)	C11—C12—C14—C15	6.7 (4)
C2—C1—C6—C7	−176.9 (2)	C12—C14—C15—C16	43.5 (4)
Cl2—C1—C6—C7	1.9 (3)	C12—C14—C15—C20	−140.1 (3)
C4—C5—C6—C1	−2.8 (4)	C20—C15—C16—C17	0.8 (4)
C4—C5—C6—C7	178.3 (3)	C14—C15—C16—C17	177.5 (2)
C1—C6—C7—C8	145.0 (3)	C15—C16—C17—C18	0.0 (4)
C5—C6—C7—C8	−36.1 (4)	C16—C17—C18—C19	−0.5 (4)
C6—C7—C8—C13	179.4 (2)	C16—C17—C18—Cl3	179.7 (2)
C6—C7—C8—C9	−2.0 (4)	C17—C18—C19—C20	0.3 (4)
C7—C8—C9—C10	−161.0 (2)	Cl3—C18—C19—C20	−180.0 (2)
C13—C8—C9—C10	17.6 (3)	C18—C19—C20—C15	0.6 (4)
C8—C9—C10—C11	−53.5 (3)	C18—C19—C20—Cl4	179.8 (2)
C9—C10—C11—C12	64.1 (3)	C16—C15—C20—C19	−1.1 (4)
C10—C11—C12—C14	138.1 (3)	C14—C15—C20—C19	−177.7 (2)
C10—C11—C12—C13	−38.5 (3)	C16—C15—C20—Cl4	179.69 (18)
C14—C12—C13—O1	7.0 (4)	C14—C15—C20—Cl4	3.1 (3)

### Hydrogen-bond geometry (Å, °)

**Table:**

*D*—H···*A*	*D*—H	H···*A*	*D*···*A*	*D*—H···*A*
C7—H7A···Cl2	0.93	2.74	3.055 (2)	101
C7—H7A···O1	0.93	2.33	2.732 (3)	105
C14—H14A···O1	0.93	2.39	2.759 (3)	103
